# Knowledge among the rural parents about the vaccinations and vaccination coverage of children in the first year of life in Papua New Guinea – analysis of data provided by Christian health services

**DOI:** 10.1186/s12879-021-05824-2

**Published:** 2021-01-30

**Authors:** Ewelina Gowin, Jerzy Kuzma, Danuta Januszkiewicz-Lewandowska

**Affiliations:** 1 Health Promotion Department, Poznan University of Medical Sciences, Fredry 10, 61-701 Poznan, Poland; 2 Faculty of Medicine and Health Sciences, Divine Word University, Madang, Papua New Guinea

**Keywords:** Vaccinations, Rural parents, The coverage rate

## Abstract

**Abstract:**

Knowledge among the rural parents about the vaccinations and vaccination coverage of children in the first year of life in Papua New Guinea – analysis of data provided by Christian Health Services.

**Background:**

This analysis aimed to assess rural parents’ knowledge about the diseases prevented by vaccinations and establish vaccination coverage in PNG.

**Methods:**

Knowledge of vaccinations was checked through a standard questionnaire (five closed questions).

We analyzed data on vaccination coverage from 2016 to 2018 from all Catholic health facilities. Analyzed vaccinations were the pentavalent vaccine (DTaP-HiB-HepB) and measles vaccine given in the first year of life. Coverage was calculated based on the number of vaccines used compared to the number of eligible children.

Analyzed vaccinations were the pentavalent vaccine (DTaP-HiB-HepB) and measles vaccine given in the first year of life.

**Results:**

Fifty-six parents, including 52 mothers and four fathers, participated in the interview. Many parents (46%) understood that the vaccine prevents diseases. During the analyzed period, 25,502 doses of measles vaccine were given, 31,428 children were vaccinated with the pentavalent vaccine. In 2016, the measles vaccine coverage rate was 26.6 and 33.4% for the pentavalent vaccine. In 2017, measles and pentavalent vaccines’ coverage rate was 12.5 and 16.6%, respectively. There were significant differences in immunization coverage between provinces. A decreasing trend in the number of administered vaccinations was observed.

**Conclusion:**

The results of this analysis demonstrate that in PNG, the majority of children are not fully immunized.

There are significant differences in the vaccination coverage between provinces. As protection from diseases is low, there is a very high risk of an outbreak of the vaccine-preventable disease in the community.

Delivery of vaccinations in PNG encounters many barriers, from access to healthcare services to natural disasters and inter-tribial conflicts.

**Supplementary Information:**

The online version contains supplementary material available at 10.1186/s12879-021-05824-2.

## Background

Papua New Guinea (PNG) has a population of around 8 million people, with a birth cohort estimated at 200,000 children [1, 2]. This is a country where vaccine-preventable diseases are still a problem. In 2017, according to Annual Report on Child Morbidity and Mortality in PNG, there were 11 cases of tetanus (four deaths), 25 cases of whooping cough, 28 cases of acute flaccid paralysis (three deaths) and three cases of measles [3]. Recent cases of polio just reminded us of this and drew attention to that unresolved problem.

In PNG, the immunization program was launched in 1977, providing vaccinations against tuberculosis, polio, diphtheria, pertussis, and tetanus. In 1981, the Expanded Program on Immunization (EPI) was started. In 2009, the pentavalent vaccine replaced the tetravalent vaccine used in 2007 and 2008. The pneumococcal vaccine was introduced in 2013. The GAVI supports the introduction of the new vaccines. Vaccinations supported in PNG by GAVI are measles, polio, pneumococcal and pentavalent vaccines [4].

The Family Health Unit organizes immunization services in PNG in the Health Improvement Branch of the National Department of Health. They are offered as a part of public health services through 800 Maternal and Child Health (MCH) clinics. Provincial Cold Chain Logistics Officers (PCCLO) are responsible for managing vaccines at a regional level with support from the Provincial Family Health Coordinator. At the district level, EPI is managed by the District Manager through the health facility (Maternal & Child clinics and Well Baby clinics) Sister-In-Charge. It covers 30% of children; the rest of the children are reached through outreach services. There are 29 outreach clinics for every 1000 children under the age of 5 years [2]. Approximately 63% of PNG health facilities are government-owned, and religious organizations organize the remaining. Church organizations offer a significant proportion of immunization services, and 99% of the population declares the Christian religion [1, 2]. The people targeted by EPI include those in the first year of life, children entering and leaving school (age 6 and 13 years), and pregnant women. The vaccination schedule in PNG is presented in Table 1.

**Table 1:** Vaccination schedule in PNG

Vaccine	Age at administration
BCG *(Bacillus Calmette–Guérin, vaccine against tuberculosis)*	birth
HepB *(vaccine against hepatitis B)*	birth
DTP Hib HepB (*vaccine against diphtheria, tetanus, pertussis, Haemophilus influenzae type B, and hepatitis B)*	1, 2, 3 months
IPV *(inactivated poliovirus given by injection)*	3 months
MR *(vaccine against measles and rubella)*	6, 9, 18 months
OPV *(weakened poliovirus given by mouth)*	1, 2, 3 months
Pneumococcal *(vaccine against Streptococcus pneumoniae)*	1, 2, 3 months
TT *(Tetanus toxoid vaccination)*	7, 13 years

The National Health Information System does monitoring of vaccination. It is difficult to establish actual vaccination coverage due to the absence of a recent coverage survey. There are different types of data provided by the government (Sector Performance Annual Review), the World Health Organization (WHO), and UNICEF [5, 6].

Available data from the National Health Information System, WHO, and UNICEF estimates are presented in Table 2. Differences between data make it very difficult to analyze it and share it with the provinces. All reports indicate a decrease in vaccination coverage, with a simultaneous dynamic increase in the number of inhabitants in the last 5 years [5–7]. The aim of this analysis was to assess knowledge among rural parents about the diseases prevented by vaccinations and to establish vaccination coverage in PNG.

**Table 2:** Vaccination coverage in PNG in years 2014–2017

Vaccine	Data source	2014	2015	2016	2017
DTP	National data	61%	54%	44%	51%
	WHO/UNICEF estimates	73%	73%	72%	62%
	Our Survey	–	–	33.4%	16.6%
Measles	National data	65%	60%	51%	43%
	WHO/UNICEF estimates	84%	79%	70%	62%
	Our survey	–	–	26.6%	12.5%

## Material and methods

It was a cross-sectional study performed by a healthcare worker in villages in 2018/2019. One healthcare worker collected data during a routine visit in villages in Simbu Province (Kervagi district) and Morobe Province (Finschhafen district). During visits in every household, a healthcare worker identified all children younger than 5 years. Parents of those children were the potential participants. To be eligible for recruitment into the survey, the caregivers were required to consent, live in Simbu or Morobe Province, and have children who were younger than 5 years. Only families who agreed to participate in the study were asked about vaccination status.

Knowledge on vaccinations was checked through an interviewer-administered questionnaire. The instrument collected socio-demographic information of caregivers, knowledge of immunization, knowledge about vaccine-preventable diseases, history of vaccines received by the child. Due to a high illiteracy rate, data were collected by face-to-face, interview-based questionnaire. Most of the questions were close-ended. The questionnaire was available in two languages English and regional language, TOKPISIN. The questionnaire was constructed for this study based on available literature on attitudes to vaccinations, and has not been published. It was validated by a group of students of the Divine Word University.

Vaccination status was checked based on vaccination records or checking the presence of a BCG scar. Parents were asked to bring a vaccination booklet, if available. Each child’s vaccination record was checked against the recommended EPI immunization schedule.

The surveyor received 1 day of training, covering an overview of research methods, interview strategies, and ethical considerations. Data obtained from questionnaires were uploaded to excel forms (no personal data were introduced to the system). The questionnaire is included as a supplementary file.

Analysis of vaccination coverage was based on the data provided by CHS from 2016 to 2018. Each year the population of children younger than 1 year covered by CHS was around 50,000, which is ¼ of the birth cohort. The target population was based on data from the Census. The CHS of PNG is the organization that represents all Christian Churches that provide health care service throughout PNG. CHS gets its funding from the Government of PNG through the National Department of Health. CHS is responsible for managing all 29 church-run health agencies within PNG. There are 713 healthcare facilities (hospitals, urban clinics, health centers, aid posts) in 22 provinces. We analyzed data on vaccination coverage from 2016 to 2018 from all Catholic health facilities that provide vaccinations in all PNG provinces. Analyzed vaccinations were the pentavalent vaccine (DTaP-HiB-HepB) and measles vaccine given in the first year of life.

### Patient and public involvement

The researchers did not assume any significant risk to themselves and the participants; the data are not of a sensitive category. We adopt an implied consent, which assumes that when, following the study’s information, the participant agrees to self-administer the questionnaire or participate in the interview, he/she agrees to take part in the survey. An informed verbal consent was obtained from all participants. Being aware of the high illiteracy, the ethics committee approved this procedure. The study received the approval of the ethics committee of the Divine Word University.

## Results

A total of 58 caregivers were eligible for the study; 56 parents, including 52 mothers and four fathers from two areas: Simbu Province (Kervagi district) and Morobe Province (Finschhafen district), participated in the interview. The response rate was 96.55%. The parents’ education level was very low: six had never been to school, 17 was four o less grade, 29 have 5–8 grade, seven have 9 or 10 classes completed. The average number of children in families was 2.5 children. The age range for participating children varies between 9 months and 9 years, while participating children’s average age was 3.3 years. Thirty-eight out of 56 parents (68%) have no vaccination records in the child’s health book.

Many parents (26 out of 56; 46%) understood that the vaccine prevents diseases. Other single opinions were that “vaccination can prevent a child for a disability” and “help a child to grow well” Almost all the women (50 out of 56) admitted to having no idea how a vaccination works. Only a few women mentioned that vaccination: “allow a child to grow” or “strengthen the child.” Seventy percent thought that the reason for vaccination is to prevent the sick (40 out of 56) or even death. To persuade other parents to vaccinate their child, the highest proportion (38%, 21 out of 56) will inform that vaccination “prevent a child from becoming sick” or from death. Others will provide a more general statement that vaccinated children “grow well.” Knowledge of diseases that can be prevented by vaccinations was shallow. The most frequent known disease was polio 18 people, and tuberculosis (TB) 15. Other conditions were tetanus and hepatitis B mentioned by four parents. The average number of known diseases preventable by vaccination was 0.8 (details in Tables 3 and 4).

**Fig. 1:**
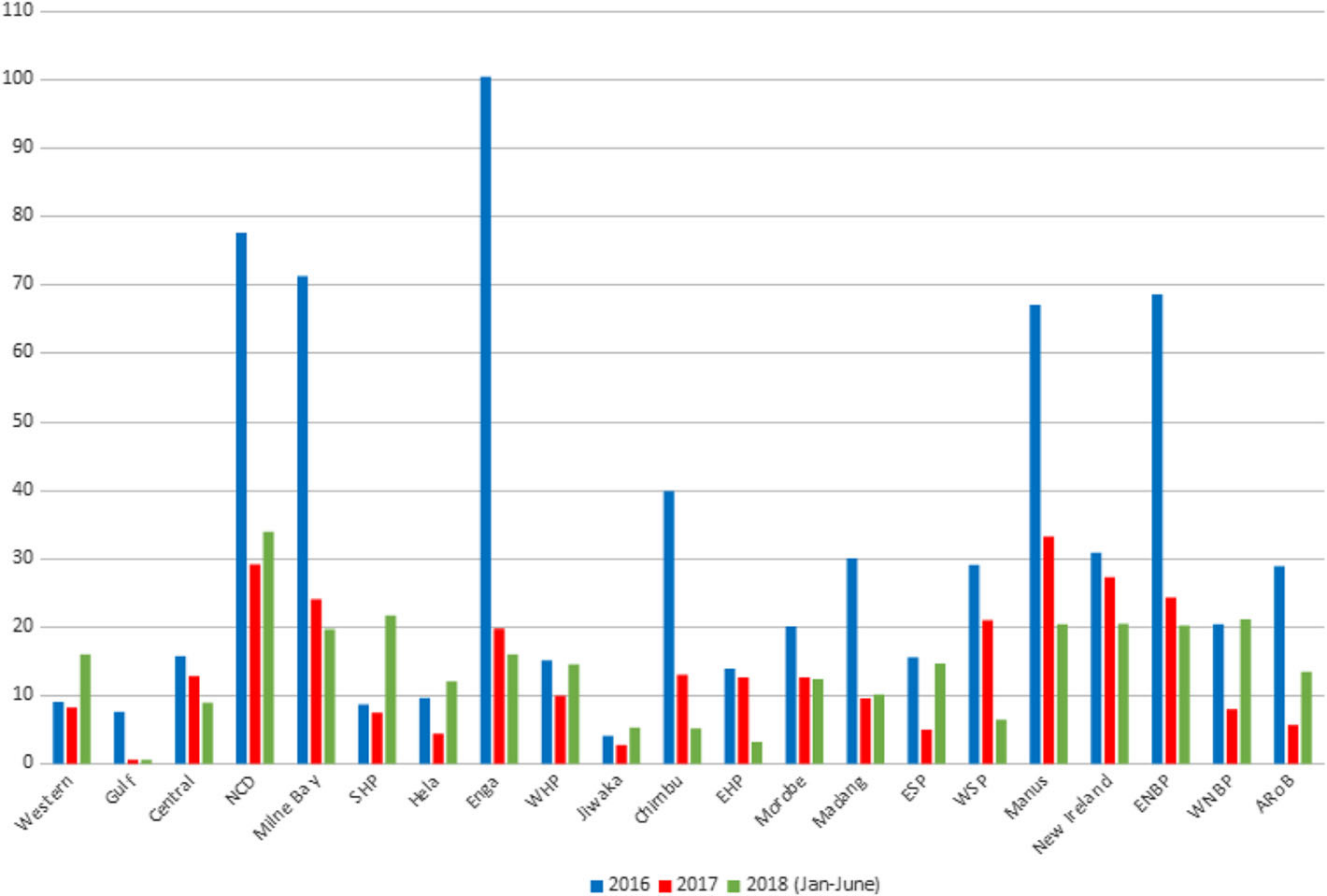
Vaccination service utilization in the clinics

**Fig. 2:**
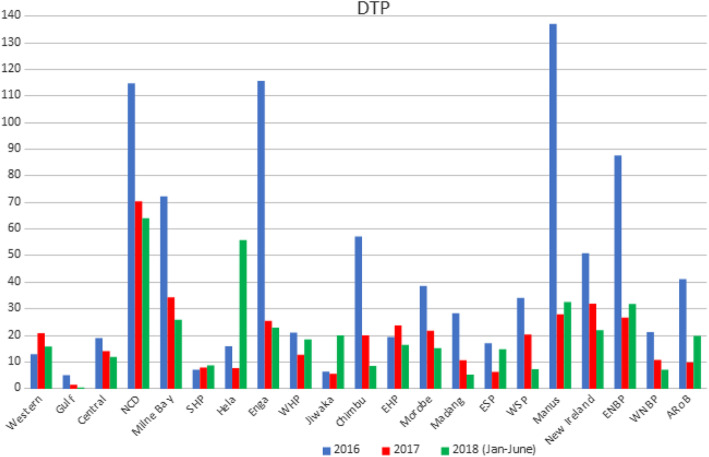
Percentage of three doses of pentavalent and measles coverage in children under 1 year of age in all provinces of PNG

During the analyzed period, 31,428 children were vaccinated with the pentavalent vaccine, and 25,502 doses of measles vaccine were given. In 2016, the measles vaccine coverage rate was 26.6 and 33.4% for the pentavalent vaccine. In 2017, measles and pentavalent vaccines’ coverage rate was 12.5 and 16.6%, respectively. There were significant differences in immunization coverage between provinces (Fig. 1). In 2016, greater than 80% coverage was noted in one province for the measles vaccine and four provinces for the pentavalent vaccine. In 2017, in none of the provinces, the coverage rate, neither for pentavalent nor for measles vaccine, was higher than 80% (Fig. 2). A decreasing trend in the number of administered vaccinations was observed.

Eighteen children had a vaccination booklet; half of them had one vaccination visit. The commonest given vaccine was DTP (diptheria, tetanus, pertussis), followed by measles and rubella vaccines given in 16 patients. Details are presented in Table 3.

**Table 3:** Results of the parents survey

Number of parents (*n* = 56)	
List, what diseases can be prevented by vaccination?	
Polio	18
TB	15
tetanus	4
hepatitis B	4
pneumonia	3
What is the role of vaccination for the child?	
“the vaccine prevents the child from the sick” “vaccination can prevent a child from disability” “help a child to grow well”	
Any idea how vaccination is working?	
no idea “allow a child to grow” “strengthen the child”	
Why it is important to vaccinate your child?	
“to prevent the sick” “to prevent the death”	
What would you tell if you like convince your neighbor to take child for vaccination?	
“vaccination prevent a child from becoming sick” “vaccination prevent a child from death” “don’t know”

**Table 4:** Vaccination status based on child’s book and observation from the rural setting of Papua New Guinea, 2019 (*n* = 18)

	Number of patients (*n* = 18)	Percentages (%)
One vaccination visit	9	50.00%
Two vaccination visits	3	16.67%
Three vaccination visits	6	33.3%
Measles vaccine	16 (8 1dose; 6–2 doses, 2–3 doses)	88.89%
Mumps vaccine	0	
Rubella vaccine	16 (8 1dose; 6–2 doses, 2–3 doses)	88.89%
Varicella vaccination status	0	
Te De Pe (DTP) vaccinated	17	94.44%
Hepatitis B vaccination x1dose	16	88.89%
Hepatitis B vaccination × 2 doses	5	27.78%
Hepatitis B vaccination × 3 doses	1	5.55%
Polio Oral vaccination ×1 dose	15	83.33%
Polio Oral vaccination ×2 doses	7	38.89%
Polio Oral vaccination ×3 doses	6	33.3%
BCG scar present	18	100.00%

## Discussion

Our analysis showed large differences between vaccination coverage in each province. In some medical facilities, the vaccination coverage was even more than 100%. This does not mean that some children were vaccinated twice, but that there were more children than before. It was a case in 10 health facilities in three provinces. The lowest coverage was in rural regions. At least 60% of the population lives in areas not accessible by road. Access to services can be, in these provinces, the biggest problem in vaccine delivery. The vaccination coverage in only one province (Morobe) is equal to median vaccination coverage in PNG. Moreover, one province (Chibu) within the analyzed period experienced a marked decline in vaccination rates – to rates lower comparing to the country average.

In PNG, there is often only one static clinic per week at the health center level, resulting in long waiting times for services. Although vaccinations are free of charge, some health facilities require user fees to run the operational costs. This discourages attendance at clinics unless the child is sick. It is proved by a mean number of outpatient visits per person per year, which in PNG is 1.28 [7]. Neonatal mortality and under 5-year mortality are among the highest in this region of the world (57/1000 live births) [1, 2, 7]. Services provided by the healthcare facilities also experience many troubles: lack of vaccinations, 30% of healthcare facilities are experiencing problems with vaccinations supply or issues with maintaining cold chain [7]. The other problem is a suboptimal number of healthcare professionals. According to the official data in PNG, there are 32 pediatricians, 0.5 physicians per 10,000 population, and 5.3 nurses per 10,000 people [2].

PNG has a relatively low coverage of essential services (Universal Health Coverage) according to WHO [6]. Antenatal care is an indicator of access to and use of health care during pregnancy, and its low use is one of the well-known risk factors for incomplete vaccination. Mean antenatal care use in PNG is estimated at 54% [8]. It varies between provinces, from 30% in Jiwaka to 98% in NCD [7]. In regions with the lowest use of antenatal care, CHS’s vaccination coverage was also low. In a study conducted by Russo in Cameroon, children born at health facilities had a higher immunization coverage rate than those born at home [9]. The percentage of supervised deliveries in PNG is estimated at 37% [7].

The other problem is the lack of public understanding of the need for vaccinations [10, 11]. There are considerable difficulties in communication. The adult literacy rate is estimated at 63.4% [1]. So far, no opposing opinions about vaccinations have been noticed in PNG, but the understanding of an idea of vaccinations is poor. For some people, there is no difference between vaccination – prevention and treatment. They view injection as a treatment. The expected benefit of participation in the survey can be raising awareness of vaccinations and their importance in protecting children and adults.

Natural disasters and military conflicts also cause difficulties in access to health services.

On 26 February 2018, the earthquake took place in four provinces Hela, Southern Highlands, Western Province, and Enga. Five hundred fourty-four thousand people were affected (46% children; 17,419 children in age 0–12 months). This natural disaster was then followed by inter-communal fighting in Hela Province.

This also caused considerable problems in vaccination. Out of 86 health facilities,18 were severely damaged. UNICEF estimated that only 10% of the target population (children younger than 5 years) received pentavalent and measles, rubella vaccination in this province [12].

According to the National Health Information System, the measles vaccine coverage in 2016 was 51% [7]. None of the provinces reported over the target 80%. The proportion of districts reporting less than 50% DTP3 (all three doses of vaccine) coverage was as high as 60%. Only 8% of provinces reported vaccination coverage greater or equal to 90%.

Data concerning the vaccine coverage obtained from CHS are much lower than the official ones. Data recording and reporting in health facilities was a shortcoming identified earlier by researchers performing studies in PNG [13–15]. Wiesen, in 2014 in a study on assessing the hepatitis B birth dose vaccination program in PNG, found out that only 17% of the health facilities were able to provide a vaccination coverage figure [13].

A lack of reliable demographic data also causes the problem with an estimation of the vaccination coverage. In PNG, birth and death registration systems are not yet sufficiently developed to accurately estimate a birth cohort. The population is growing very fast; hence, an entire birth cohort seems to be more significant. The latest polio epidemic showed that the pediatric population might be more significant than assumed. In the first round of catch-up vaccination action covering the three high-risk provinces of Morobe, Madang, and the Eastern Highlands, the estimated number of children was 289,582, but 303,907 (105%) children under 5 years old were vaccinated [16, 17]

A field survey is another way to obtain vaccination coverage. It can be done by checking patients’ vaccination records. But in PNG, the so-called baby book is missed very often. In a survey conducted by Samiak, according to the medical records of 70 patients and based on the interview with parents, only 15% of children had complete vaccination status [11]. In our analysis, only 18/56 had patients had baby books; 68% had no vaccination records. Based on vaccination records, half of the patients had only one vaccination visit.

The main limitation of the study is a small sample of questioned parents. Taking into consideration local customs, it is challenging to schedule extensive studies in PNG. Indigenous people are very wary of contact with strangers.

There is a shortage of healthcare professionals in PNG, and because of their workload, they are not very eager to be involved with surveys. So this is why we should have accepted this small study as the only possible way of gaining insight into PNG vaccination practices. The small sample can bias our data. We are aware that this may not be representative of the whole population. Parents living in big cities may have different opinions on vaccinations. But the vast majority of people in PNG live in villages, and most of the children are born there.

Second, only written records (vaccination cards) were accepted. It can cause some inaccuracies in vaccination history. It is possible that some more doses were given. But because of a high illiteracy rate, it is not possible to rely on parents’ reports.

When it comes to data on vaccination coverage – CHS covers with their services around ¼ of the birth cohort in all the provinces, so the analyzed data are representative for PNG.

Improving vaccination coverage in PNG is essential for outbreak control. To achieve this, it is necessary to improve the quality of services delivered by healthcare facilities and increasing community awareness of the role of vaccinations. As observed in our analysis, declining coverage rates are in accordance with data provided by GAVI, WHO, and Country Official estimates. This is a very worrisome trend.

## Conclusion

The results of this analysis demonstrate that in PNG, the majority of children are not fully immunized. As protection from diseases is low, there is a very high risk of an outbreak of the vaccine-preventable disease in the community.

There are significant differences in vaccination coverage between provinces. This can be improved by a better distribution of healthcare services, especially in rural areas.

The problem is a lack of public understanding of the need for vaccinations – this can be improved by media campaigns oriented to inform people about the benefits of vaccinations.

Lack of patient vaccination records makes it difficult to establish individual vaccination history.

There is a need for a central registry, where each given dose of vaccine will be recorded.

## Supplementary Information

**Additional file 1:.** Questionnaire TOKPISIN RURAL. Questionnaire in English and TOKPISIN.

## Data Availability

The datasets used during the current study are available from the corresponding author on reasonable request.

## Abbreviations

AroB: Autonomous region of Bougainville
BCG: Bacillus calmette–guérin
DTP: Diptheria, tetanus, pertussis
EH: Eastern highlands
ENB: East New Britain
EPI: Expanded Program on Immunization
ES: East sepik
HepB: Hepatitis B
Hib: *Haemophilus influenzae* type B
IPV: Inactivated poliovirus vaccine
MR: Measles, and rubella
MCH: Maternal and child health clinics
NCD: National capital district
OPV: Oral poliovirus vaccine
PCCLO: Provincial cold chain logistics officers
PNG: Papua New Guinea
SH: Southern Highlands
TB: Tuberculosis
TT: Tetanus toxoid vaccine
WH: Western highlands
WHO: World health Organization
WNB: West New Britain
