# Supplementary material for: Knowledge among the rural parents about the vaccinations and vaccination coverage of children in the first year of life in Papua New Guinea – analysis of data provided by Christian health services
Source: BMC Infect Dis. 2021 Jan 30;21:130. doi: 10.1186/s12879-021-05824-2 (PMC7847142; doi:10.1186/s12879-021-05824-2)
Supplement: Supplementary file 1 — Additional file 1:. Questionnaire TOKPISIN RURAL. Questionnaire in English and TOKPISIN. [file 12879_2021_5824_MOESM1_ESM.docx]

**`QUESTIONNAIRE – Vaccination status of children under 10 in the rural PNG**
DOB (**Dei pikinini i bin bon**) ______ or Age (**Krismas**) ___Sex (**Man/Meri**)___ From pregnancy no.(**Namba bilong pikinini insait long femili**)____ No of children in family(**Hamaspela pikinini insait long femili**?)___________

Children's health book present yes _______no___________

**Vaccinations checked on the basis of Children's health book**Vaccinated: once (**wanpela taim**)________ twice (**tupela taim**) _________3 times (**tripela taim**) _________

|  | 1 dose | 2 dose | 3 dose |
| --- | --- | --- | --- |
| DTP diphtheria / tetanus / pertussis |  |  |  |
| DTP-HiB-Hepatitis B |  |  |  |
| Polio (IM) |  |  |  |
| Polio (oral) |  |  |  |
| Hepatitis B |  |  |  |
| Measles |  |  |  |
| Rubella |  |  |  |
| Pneumoccocal vaccine |  |  |  |
| BCG |  |  |  |

**Vaccinations based on conversation with child’s mother**

|  | 1 dose | 2 dose | 3 dose |
| --- | --- | --- | --- |
| DTP diphtheria / tetanus / pertussis |  |  |  |
| DTP-HiB-Hepatitis B |  |  |  |
| Polio (IM) |  |  |  |
| Polio (oral) |  |  |  |
| Hepatitis B |  |  |  |
| Measles |  |  |  |
| Rubella |  |  |  |
| Pneumoccocal vaccine |  |  |  |
| BCG |  |  |  |

Date:of completing questionnaire (**Dei bilong wokim kwestene**?) _________________

Part B Knowledge & attitude

Guardian Mother (**Was mama**)_______ Father (**Papa**)__________ Age (**Krismas**)______

Education (**Skul/Greid**)___________________________

Job (**Wok**)­­­­­­­­________________________________

List, what diseases can be prevented by vaccination?

**Kisim sut bilong banisim sik (bebi sut) em inap halivim long stopim ol wanem kain sik?**

What is the role of vaccination for the child?

**Long wanem as pikinini i mas kisim bebi sut?**

Any idea how vaccination is working?

**Yu i gat sampela tingting long hau bebi sut I sawe wok?**

Why it is important to vaccinate your child?

**Long wanem as pikinini i mas kisim bebi sut?**

What would you tell if you like convince your neighbor to take child for vaccination?

**Sapos yu i laik grisim neiba o olain ol i stap arere long yu long kisim pikinini i go kisim bebi sut, bai yu I tok wanem long ol?**


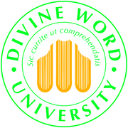


**The Executive Officer to the Dean of FMHS** **Faculty of Medicine & Health Sciences**
**Phone:** (+675) 422 2937 **Ext:** 770 **Fax:** (+675) 422 2812 **Email:** [jpais@dwu.ac.pg](mailto:jpais@dwu.ac.pg)
